# Targeted Physiotherapy for an Interesting Case of Spontaneously Resolving Extracapsular Infarct: A Case Report

**DOI:** 10.7759/cureus.52348

**Published:** 2024-01-16

**Authors:** Neha P Arya, Nikita H Seth, Ghanishtha C Burile, Raghumahanti Raghuveer

**Affiliations:** 1 Neurophysiotherapy, Ravi Nair Physiotherapy College, Datta Meghe Institute of Higher Education and Research, Wardha, IND

**Keywords:** physiotherapy, case report, strength, gait, balance, rehabilitation, ischemic stroke, external capsule

## Abstract

Stroke is a prevalent and disabling illness that is becoming more common in developing countries. After a stroke, physical inactivity frequently results in long-term deconditioning and disappointing consequences. This case study focuses on an infrequent 0.3% of ischemic stroke cases that occur in the external capsular (ECC) or extreme capsular (EXC) region. In sub-insular infarcts, ECC-EXC lesions are distinct and frequently linked to the anterior opercular syndrome. We are presenting the case of an 86-year-old female patient who had a fall and loss of consciousness. Diagnostic tests revealed that the patient had an extracapsular ischemic event; due to unstable vital signs and frequent drop in saturation of peripheral oxygen (SpO2) levels, the patient was intubated and admitted to the intensive care unit (ICU). When stable, the patient experienced generalized weakness, for which she was referred for physical therapy. Balance and gait impairments were secondary to weakness. A planned two-week structured physiotherapy intervention was created with an emphasis on gait training, muscle strengthening, and balance. Adaptive gait training, progressive exercises, and balancing activities addressed the patient's limitations. This case study demonstrates how an elderly individual with an external capsule ischemic event can benefit from targeted physical therapy for increasing muscle strength, balance, and gait performance. Positive results emphasize how crucial early and targeted physiotherapy is for supporting stroke survivors' neurological recovery.

## Introduction

An abrupt neurological outburst brought on by reducing blood vessel perfusion to the brain is referred to as stroke, one of the most common debilitating illnesses affecting adults [1]. Between 1970 and 1979 and 2000 and 2008, the incidence of stroke increased by more than 100% in low- and middle-income countries, including India [2,3]. Age also has an impact on the incidence of stroke in both men and women. The incidence rises slightly with age in men, but it is higher in women at younger ages. In stroke patients, ischemic occlusions account for about 85% of deaths, with intracerebral haemorrhage accounting for the remaining 15%. In the brain, ischemic occlusion causes thrombotic and embolic conditions [4,5]. Regretfully, over 20% of stroke victims never learn to walk independently, and even those who do rarely find it easy to get around the neighbourhood. Because of these persistent issues, physical inactivity will lead to deconditioning and unsatisfactory long-term outcomes [6-8].

The external capsular (ECC) or extreme capsular (EXC) region was rarely the site of an ischemic stroke. In individuals with sub-insular infarcts who had anterior opercular syndrome, the ECC or EXC involvement can often be seen. Which, according to our registry, corresponds to 0.3% of our ischemic stroke patients [9,10]. This tiny region was primarily affected by the insular cortical infarct or the nearby large lenticulostriate infarcts. The ECC is a band composed of longitudinally coursing fibres flanked by the claustrum, a grey matter plate whose connections and purpose are unknown. The extreme capsule, a layer of subcortical white matter, lies between the insula and the claustrum [11-13]. Corticotegmental fibres that originate from the frontal, temporal, anterior parietal, and insular cortexes and travel caudally to the midbrain segmentum via a sublenticular pathway may be the most significant elements of the external capsule [14]. The ECC-EXC lesion was discovered to be located where the lenticulostriate artery branches and tiny, insular penetrating arteries are encountered. There is some data on the clinical picture of ECC-EXC lesions in the literature, but they are limited to cases where the lesion extends to the caudate nucleus or the lenticular lesion. The primary clinical observation of ECC-EXC infarct was mild motor impairment of the faciobrachial or faciobraciocrural types [15-17].

## Case presentation

An 86-year-old female experienced a fall and loss of consciousness while working at home; she was taken to hospital by her relatives, and after examination, the patient was unresponsive and vitally unstable. She was placed on a mechanical ventilator synchronized intermittent mandatory ventilation (SIMV) mode and intubated due to a persistent decline in her saturation of peripheral oxygen (SpO_2_) levels. The patient had a history of hypertension for six years and diabetes mellitus for three years. Specific investigations like computed tomography (CT) scans and Magnetic Resonance imaging (MRI) were done. Following the patient’s transfer to the intensive care unit (ICU), anti-platelet medication therapy using tab. clopiatab (75 mg) and tab. rosuvastatin (20 mg) was administered, and the next day, the patient was conscious; after three days, the patient maintained her SpO_2_ levels and was extubated. The patient was referred for physiotherapy. The next day, she was shifted to the ward; the patient complained of generalized weakness and difficulty in getting up from bed with the inability to walk. Physiotherapy rehabilitation was continued.

Clinical findings

Informed consent was taken from the patient’s relative before treatment; the patient was assessed supine and was vitally stable. A neurological examination was performed, muscle tone was found to be normal, and muscle strength assessment was performed according to the Modified Oxford Grading System, as depicted in Table 1. The strength of both the upper and lower limbs was reduced; all superficial, deep, and combined cortical sensations were intact. All deep tendon reflexes (DTRs) were found to be grade +1 (diminished), and the plantar reflex was seen as a flexor response. Pre- and post-rehabilitation balance and gait assessments were conducted using the Berg’s Balance Scale (BBS) and Dynamic Gait Index (DGI). Functional independence measure (FIM) was used to analyze activities of daily living (ADLs).

**Table 1:** Manual muscle testing (MMT) pre-rehabilitation 5+: Complete range of motion (ROM) against gravity with maximal resistance, 4: complete ROM against gravity with moderate resistance, 3+: complete ROM against gravity with minimal resistance, 3: complete ROM against gravity, 3-: some but not complete ROM against gravity, 2+: Initiates motion against gravity, 2: complete ROM with gravity eliminated, 2-: Initiates motion if gravity is eliminated, 1: Evidence of slight contractility but no joint motion, 0: No contraction palpated

Muscle group	Action	Left	Right
Shoulder	flexors	2+	2+
	extensors	2-	2-
	Abductors	2-	2-
	Adductors	2-	2-
Elbow	Flexors	2+	2+
	Extensors	2+	2+
Wrist	Flexors	2-	2-
	Extensors	2-	2-
Hip	Abductors	1	1
	Adductors	1	1
	Flexors	2-	2-
	Extensors	1	1
Knee	Extensors	2+	2+
	Flexors	1	1
Ankle	Plantar Flexors	2+	2+
	Dorsi flexors	2-	2-

Diagnostic assessment 

**Figure 1:**
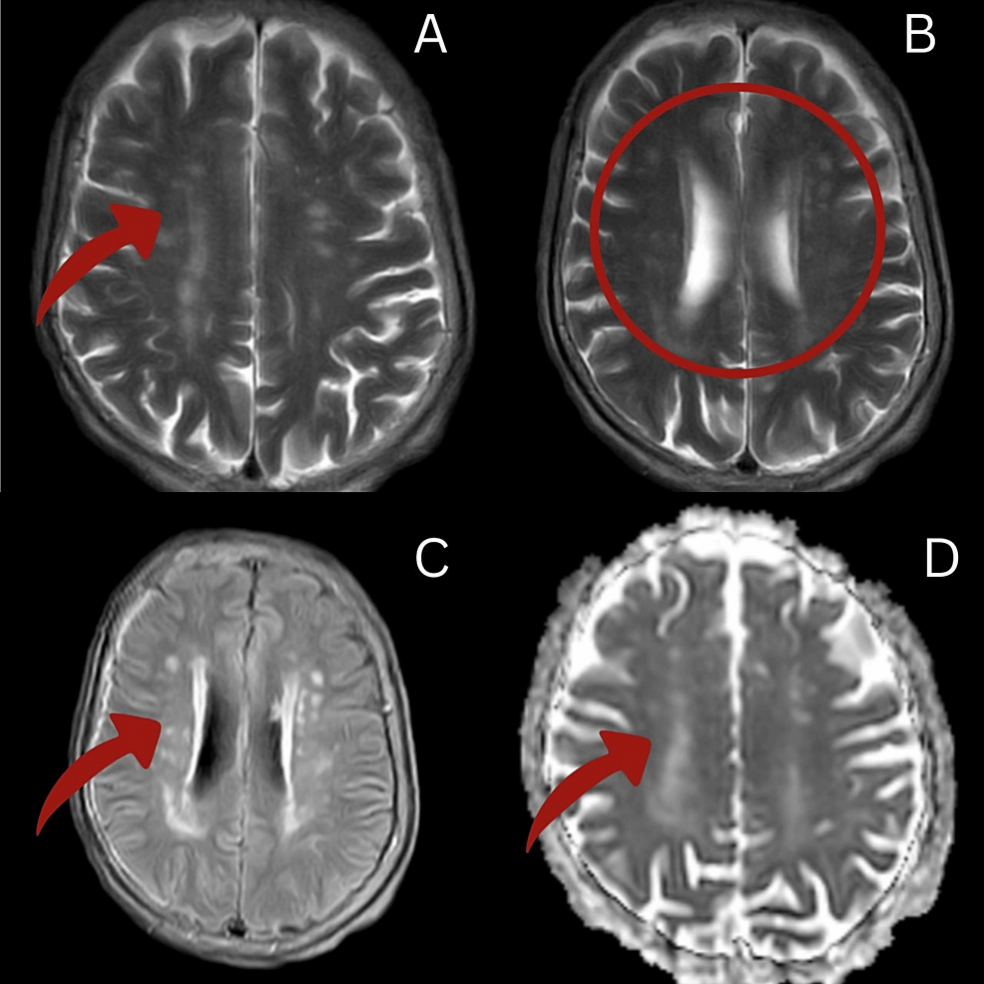
Different weighted images of MRI Brain A, B: eT2w TRA image, C: eAX FLAIR image, D: dADC image, Red arrow: showing bilateral extracapsular infarct MRI: Magnetic Resonance imaging

CT scan and MRI was done, which showed a bilateral extracapsular lacunar infarct. According to TOAST classification, this denotes small-vessel occlusion. Figure 1 depicts different weighted images of MRI.

Physiotherapy management

Physiotherapy (PT) management was planned week-wise according to the patient’s recovery; Table 2 depicts a summary of PT management for week 1. Table 3 summarizes PT management for week 2.

**Table 2:** PT rehabilitation protocol for week 1 PT: Physiotherapy, PNF: Proprioceptive Neuromuscular Facilitation, D1/2: Diagonal pattern, ROM: Range of Motion, reps: repetitions

Goals	Intervention	Dosage
To improve movement quality of the upper limb and lower limb.	Rhythmic initiation PNF D1 Flexion upper extremity, D2 Flexion lower extremity	10 reps x 2 sets
To improve shoulder girdle strength.	Active-assisted shoulder ROM	10 reps x 2 set
To improve the strength of muscles around the knee joint	Active-assisted knee extension (sitting), Active-assisted knee flexion (side-lying)	10 reps x 2 set
To enhance strength for ankle and foot muscles.	Active-assisted ROM for ankle joint	10 reps x 2 set
To enhance bed mobility and transitions.	Bed mobility exercises assisted sit-to-stand	5 reps x 1 set
To improve balance and proprioception.	Weight shifts in sitting and standing (eyes open), balancing on a stable surface	10 reps x 2 set
To enhance gait pattern.	Walking with the support of a walker	1-2 rounds
To improve trunk and pelvis stability.	Rhythmic stabilization for trunk and pelvic muscles.	10 reps x 1 set
To improve grip strength.	Sponge ball, putty compression	10 reps x 2 sets

**Table 3:** PT rehabilitation protocol for week 2 PT: Physiotherapy, PNF: Proprioceptive Neuromuscular Facilitation, D1/2: Diagonal pattern, ROM: Range of Motion, reps: repetitions, SLR: Straight Leg Raising, lbs: pounds, reps: repetitions

Goals	Intervention	Dosage
To improve muscle strength and coordination	Combination of isotonic PNF D1 Flexion-extension upper limb, D2 Flexion-extension lower limb	10 reps x 2 sets
To improve the strength of the shoulder girdle	Active ROM for shoulder Shoulder shrugs and scapular retractions.	10 reps x 2 sets
To improve the strength of the lower limb	Active SLR, Dynamic quadriceps, Hamstring curls	10 reps x 2 sets
To improve strength for ankle and foot muscles.	Resistance band exercises for ankle and foot- yellow band (3lbs)	10 reps x 1set
To improve coordination	Coordination exercises	10 reps x 1 set
To improve balance	Weight shifts in standing, tandem standing (eyes closed), multi-directional reach, balancing on unstable surface	10 reps x 1 set
To improve gait pattern	Sideways walking, walking around obstacles, walking over the obstacles.	2-3 rounds
To improve the strength of the wrist and intrinsic muscles.	Resistance band exercises for wrist and hand- yellow band (3lbs)	10 reps x 1 set
To improve fine motor activities	Zipping activity, knot-tying activity, hooking-unhooking, lock and key activity	10 reps x 1 set
To improve gross motor function	Peg-board Activity, cylindrical grasp, spherical grasp	10 reps x 1 set

**Figure 2:**
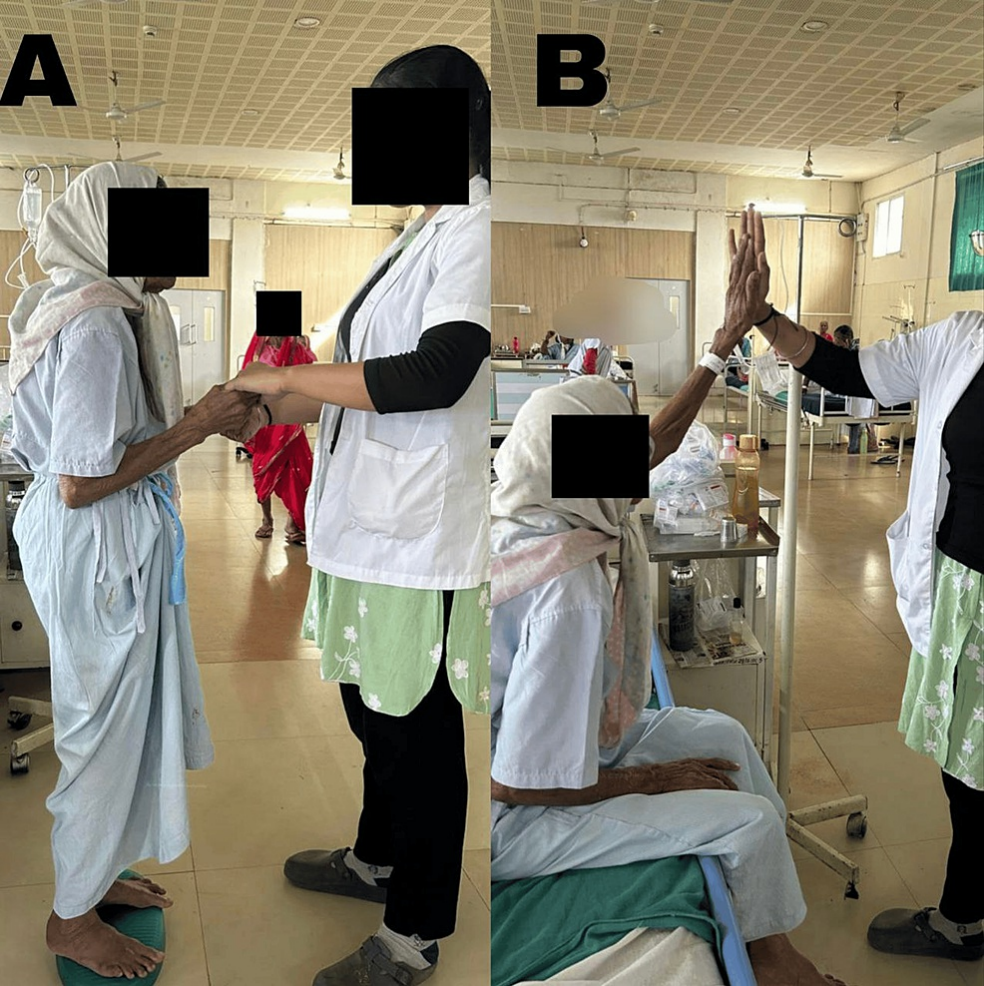
Patient performing physiotherapy exercises A: balance training on unstable surfaces, B: multidirectional reaching in a sitting position

**Figure 3:**
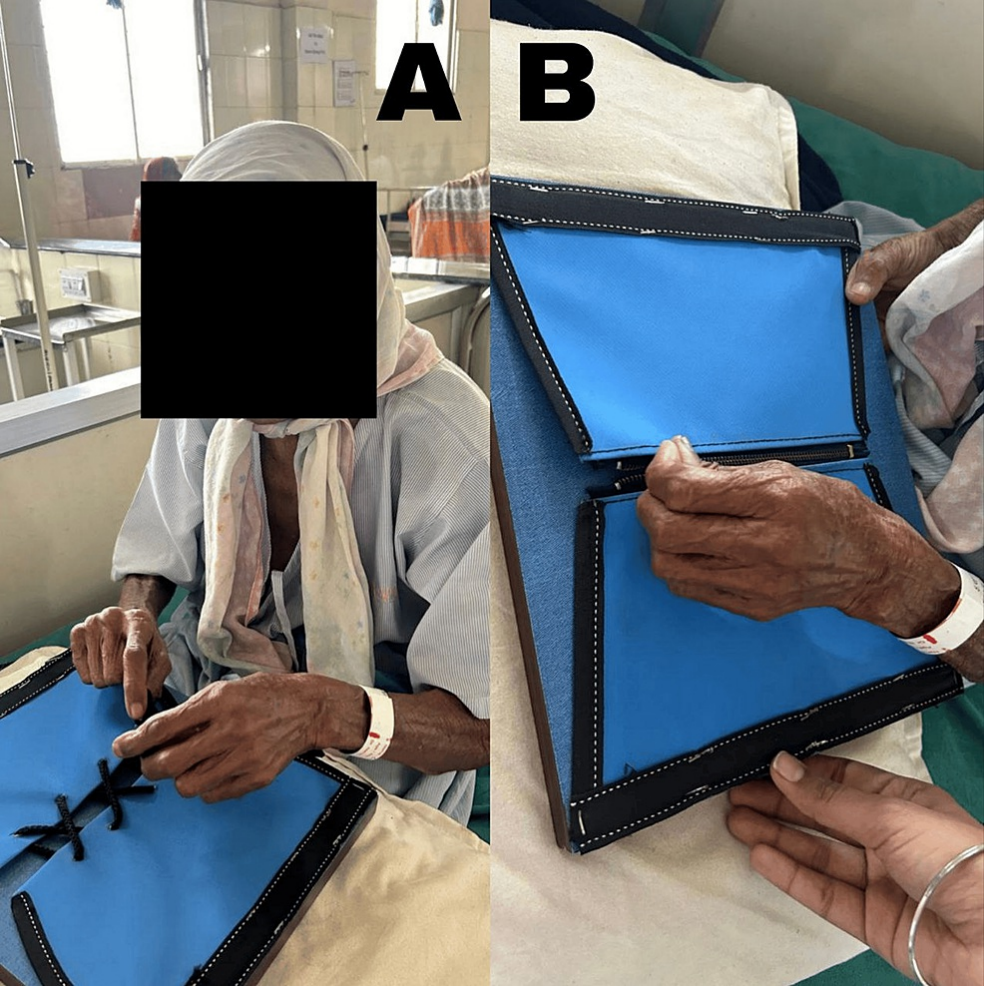
Patient performing fine motor training A: knot-tying activity, B: zipping activity

Figure 2 depicts the patient performing balance training exercises, and Figure 3 includes the patient performing fine motor training activities.

**Figure 4:**
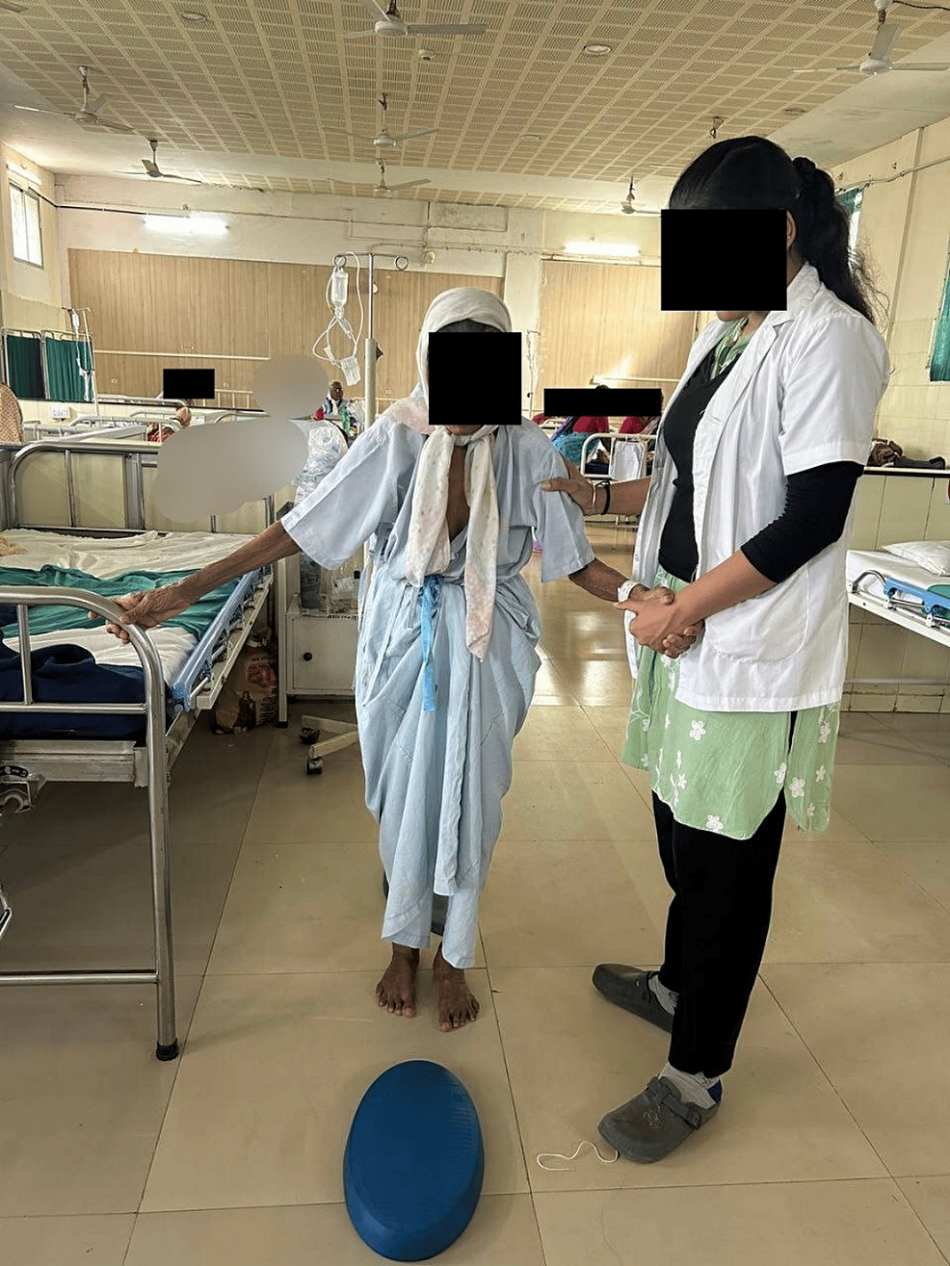
Gait training around the obstacles

**Figure 5:**
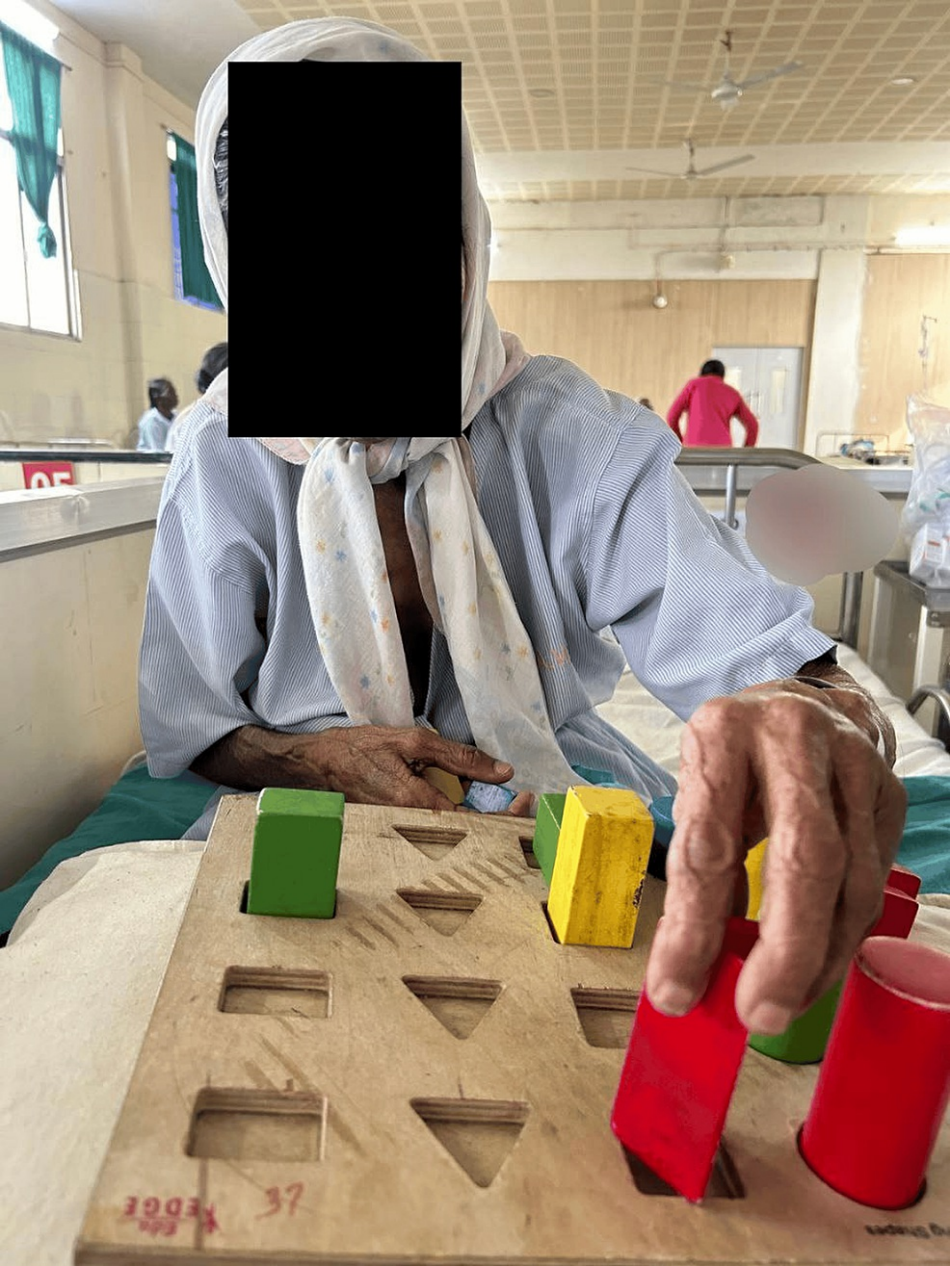
Patient performing a peg-board activity

Figure 4 depicts the patient performing the gait training activity; Figure 5 shows the patient performing the peg-board activity.

Follow-up and outcome measure

An organized physiotherapy protocol was planned for two weeks, and assessment was taken before and during treatment at the first and second weeks of the rehabilitation program. Follow-up was also taken in the sixth week; findings of outcome measures are summarized in Table 4, and manual muscle testing findings are depicted in Table 5.

**Table 4:** Pre- and post-rehabilitation outcome measures ICU: Intensive care unit

Outcome measure	Pre-rehabilitation	Week 1	Week 2	Week 6
Bergs Balance Scale	8/56	28/56	35/56	47/56
Dynamic Gait Index	0/24	4/24	13/24	19/24
ICU Mobility Scale	0/10	5/10	-	-
Functional Independence measure	18/126	86/126	98/126	118 /126

**Table 5:** Manual muscle testing (MMT) evaluation at week 1, week 2 and week 6 5+: Complete range of motion (ROM) against gravity with maximal resistance, 4: complete ROM against gravity with moderate resistance, 3+: complete ROM against gravity with minimal resistance, 3: complete ROM against gravity, 3-: some but not complete ROM against gravity, 2+: Initiates motion against gravity, 2: complete ROM with gravity eliminated, 2-: Initiates motion if gravity is eliminated, 1: Evidence of slight contractility but no joint motion, 0: No contraction palpated

Muscle group	Action	Week 1		Week 2		Week 6	
		Right	Left	Right	Left	Right	Left
Shoulder	flexors	3-	3-	3	3	3+	3+
	extensors	2+	2+	3-	3-	3+	3+
	Abductors	3-	3-	3	3	3+	3+
	Adductors	2	2	3+	3+	3+	3+
Elbow	Flexors	3	3	3+	3+	4	4
	Extensors	3	3	3+	3+	4	4
Wrist	Flexors	3	3	3	3	3+	3+
	Extensors	3	3	3	3	3+	3+
Hip	Abductors	2	2	3	3	3+	3+
	Adductors	2	2	3+	3+	3+	3+
	Flexors	3-	3-	3	3	3+	3+
	Extensors	2+	2+	3-	3-	3	3
Knee	Extensors	3	3	3+	3+	3+	3+
	Flexors	3	3	3+	3+	3+	3+
Ankle	Plantar Flexors	3-	3-	3	3	3+	3+
	Dorsi flexors	3-	3-	3	3	3+	3+

## Discussion

Stroke rehabilitation aims to improve and expedite the restoration of motor function through a blend of compensation- and restitution-focused therapeutic approaches. Brain plasticity is the foundation of restoration-oriented methods. Approaches that focus on compensation are more task-oriented. The main objective is to improve performance in primary and vital daily living activities while minimizing reliance on outside assistance and maximizing safety [18,19]. The goal is to return as many aspects of normal voluntary movement as possible, including strength in specific muscle groups and, most importantly, normal motor control. With this objective in mind, patients receive training on using their limbs as effectively as possible, which need not be natural either with or without external aids [20].

In the following case, the patient presented with generalized weakness due to a bilateral infarct in the region of the external capsule; balance and gait impairments were secondary to weakness. Detailed neurological examination was performed; pre- and post-outcome measures used were manual muscle testing (MMT), BBS, DGI, and FIM. Physiotherapy protocol was planned according to impairments, which included strengthening, bed mobility, balance activities, fine and gross motor training, and gait training; the total duration for treatment was two weeks, and assessment was done at the end of each week; follow-up was also taken at the sixth week, which significantly improved outcomes. The patient had rapid resolution of symptoms and improved muscle strength, gait pattern, and balance ability, which led to independence in ADLs. Physiotherapy plays a vital role in a patient’s recovery. The current study discovered how early rehabilitation helps in regaining early functioning.

## Conclusions

This case study demonstrates the efficacy of a comprehensive physical therapy intervention in managing the spontaneously resolving bilateral external capsule infarct in an 86-year-old patient. Muscle strength, balance, and gait performance improved dramatically during the structured two-week rehabilitation program, which increased functional independence in daily living activities. Outcome measures like the FIM, BBS, DGI, and MMT quantitatively evaluated the patient's progress. This case emphasizes how vital early and focused physical therapy is to helping stroke victims recover neurologically and regain optimal motor function, which in turn improves their general health and quality of life.
